# Regulation of Nuclear Factor Kappa-Light-Chain-Enhancer of Activated B Cells (NF-κβ) in Inflammatory Bowel Diseases

**DOI:** 10.3389/fped.2018.00317

**Published:** 2018-10-30

**Authors:** Deenaz Zaidi, Eytan Wine

**Affiliations:** ^1^Department of Pediatrics, University of Alberta, Edmonton, AB, Canada; ^2^Centre of Excellence for Gastrointestinal Inflammation and Immunity Research, University of Alberta, Edmonton, AB, Canada; ^3^Department of Physiology, University of Alberta, Edmonton, AB, Canada

**Keywords:** nuclear factor kappa-light-chain-enhancer of activated B cells (NF-κβ), inflammatory bowel diseases (IBD), Crohn Disease (CD), ulcerative colitis (UC), immunity, microbes, homeostasis

## Abstract

Inflammatory bowel diseases (IBD), encompassing both Crohn Disease (CD) and ulcerative colitis (UC) are globally prevalent diseases, impacting children of all ages. The hallmark of IBD is a perturbed immune system that leads to continuous inflammation in the gut and challenges optimal treatment. Nuclear factor kappa-light-chain-enhancer of activated B cells (NF-κβ), a nuclear transcription factor, plays a major role in gut homeostasis and contributes significantly toward a balanced, homeostatic immune system. Dysregulation in the NF-κβ pathway and factors that regulate it lead to a state of uncontrolled inflammation and altered immunity, as typically observed in IBD. Levels of proinflammatory cytokines that are regulated through NF-κβ are increased in both CD and UC. Genes known to activate NF-κβ, such as, Nucleotide-binding oligomerization domain-containing protein 2 (NOD2) and Interleukin 23 (IL-23), are associated with IBD. Factors involved in inhibition of NF-κβ, such as A20 and TOLLIP, are also affected in IBD, resulting in failed inflammation suppression/regulation. NOD-2 and A20 have specifically been found to be strongly associated with pediatric IBD. Gut commensals are known to exert anti-inflammatory activities toward NF-κβ and can have a potential role in attenuating inflammation that likely occurs due to microbial dysbiosis in IBD. Failure to terminate/downregulate NF-κβ signaling results in chronic inflammation in IBD. Well-regulated control of inflammation in children with IBD can help better control the disease and suppress immune responses. Better understanding of factors that control NF-κβ can potentially lead toward discovering targeted therapeutic interventions for IBD. Suppression of NF-κβ can be achieved through many modalities including anti-sense oligonucleotides (ASOs), siRNA (small interfering RNA), factors regulating NF-κβ, and microbes. This review focuses on the role of NF-κβ, especially in pediatric IBD, and potential therapeutic venues for attenuating NF-κβ-induced inflammation.

## Background

The immune system of the gastrointestinal tract is normally well-tuned with the gut microenvironment, which enables the existence of a steady homeostatic state. The gut environment is continuously exposed to various exogenous materials, including food, xenobiotics, and microbial pathogens. Eradication of pathogens with simultaneous survival of gut commensals that are beneficial for maintaining homeostasis is a major challenge faced by the intestinal immune system. Nature has many protective mechanisms in place that help maintain this stable environment in the gut. This stability is disrupted in disease conditions affecting the gastrointestinal system, such as inflammatory bowel diseases (IBD). IBD, including both Crohn disease (CD) and ulcerative colitis (UC) have a debilitating impact on the lives of children and adults alike (1, 2). Some of the major features distinguishing between adult and pediatric IBD are nutritional challenges, poor bone health, delayed puberty, and growth failure, all of which are linked to inflammation (3, 4). Given the complex complications of IBD in children, it is critical to understand the basic factors that trigger inflammation and modulate treatment regimens accordingly.

Although many factors have been associated with IBD, the etiology is still not clearly understood, but seems to involve integrated mechanisms of uncontrolled immune response to various environmental/microbial stimuli in genetically susceptible hosts. Recent research has highlighted the importance of nuclear factor kappa-light-chain-enhancer of activated B cells (NF-κβ) in regulating immune responses in the gut. This review will focus on the role of NF-κβ in IBD and potential therapeutic mechanisms that can control NF-κβ-mediated inflammation in IBD, highlighting aspects especially relevant to children (5).

NF-κβ, a nuclear transcription factor, is a central player of sustaining a stable state of innate immunity in the gut. Disruptions and imbalances in the NF-κβ pathway lead to chronic inflammation, dysregulation of natural immune responses (6, 7), and altered immunity in IBD (8). Pathogenesis in both CD and UC is heavily marked by expression of multiple proinflammatory cytokines (9), many of which are regulated through NF-κβ. In fact, several of the key genes associated with IBD, such as Nucleotide-binding oligomerization domain-containing protein 2 (NOD2) and Interleukin 23 (IL-23), drive NF-κβ activation. On the flip side, dysregulation of NF-κβ inhibitory pathways, such as reduced expression of A20 (tumor necrosis factor α-induced protein 3; TNFAIP3) or TOLLIP (10), could also contribute to unremitting inflammation, as seen in NF-κβ essential modulator (NEMO) epithelial cell-specific knockout mice (8, 11). Thus, better understanding of factors that drive and control NF-κβ could lead to targeted therapeutic interventions for inflammatory conditions, including IBD.

As we hypothesize that dysregulation, or the inability to terminate NF-κβ signaling is critical for the persistence of chronic inflammation in IBD, we would argue that this is especially important in children as microbe-driven persistent inflammation cannot be turned off in this setting and is likely to drive chronic inflammation at its early stages. We have recently shown that A20, a negative regulator of NF-κβ, is specifically disrupted in pediatric IBD; despite an observed increase in A20 gene expression, protein levels and associated signaling are reduced, suggesting a pediatric-specific dysregulation of A20 and NF-κβ (12). In contrast, adult studies have shown variable expression of A20 (13).

Early life factors can define the immune milieu and microbial interactions and predispose for immune-mediated conditions, such as IBD; regulation of NF-κβ is likely to be critical for this process. For all these reasons, better defining how NF-κβ is regulated in children is likely to provide important insight into pathogenesis and guide future therapies. It is quite likely that as more therapies will target NF-κβ regulation, physicians and scientist caring for children with IBD would benefit from deeper understanding of this complex pathway.

## Structure of NF-κβ

**Figure 1:**
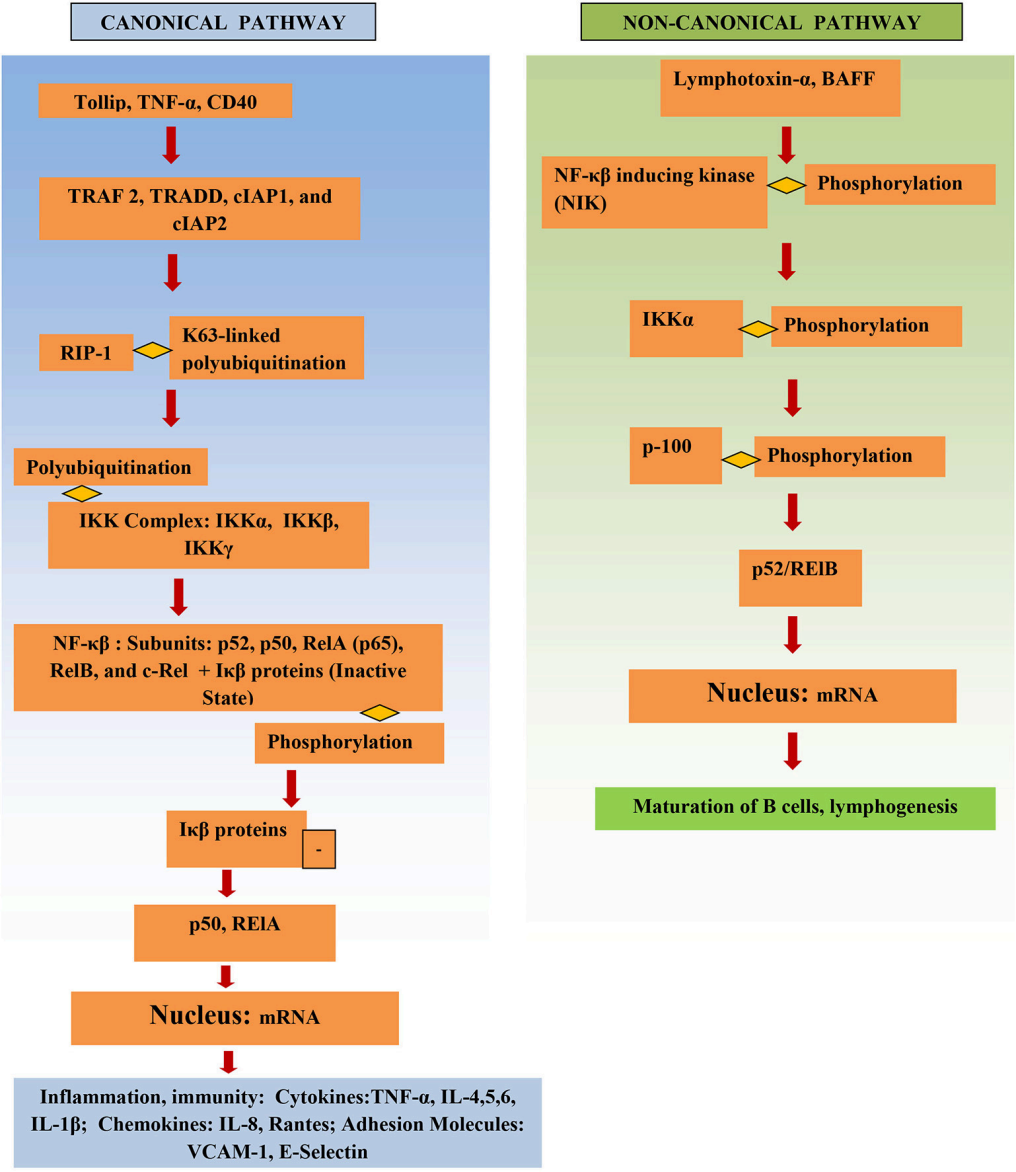
Structure and activation of NF-κβ.

NF-κβ structure consists of multiple protein subunits: p52, RelA (p65), p50, c-Rel, and RelB, which are coalesced in the cytoplasm bound to Iκβ proteins (Figure 1). N-terminal Rel homology domain (RHD) is shared by all subunits and is essential for dimer formation. The subunits are conjoined in the cytoplasm in the resting state and remain inactive while attached to Iκβ proteins. Gene transcription can be regulated by RelA, RelB, and c-Rel as they have a transcriptional activation domain (TAD). The transcriptional activity of the p50 and p52 subunits depends upon binding with proteins that have TAD, for example, RelA, RelB, and c-Rel (14).

The IKK complex is the major factor that activates the NF-κβ pathway. It consists of IKKγ (a non-catalytic protein) and the kinases IKKα and IKKβ. Phosphorylation of Iκβ by IKK results in its proteasomal degradation and thus activates NF-κβ, releasing NF-κβ, and resulting in NF-κβ subunits being translocated into the nucleus and leading to proinflammatory gene transcription.

## NF-κβ signaling pathways

The NF-κβ pathway is activated either through the canonical or the non-canonical pathway (Figure 1). Initiation of the canonical pathway occurs through a process of receptor-ligand binding. Binding and stimulation of TLRs with antigens leads adaptors, such as TRADD, TRAF 2, cIAP1, and cIAP2 and RIP1, to bind to NF-κβ receptor's cytoplasmic domain. K63-linked polyubiquitination of RIP1 aids the IKK complex in recruitment of NF-κβ to the activated receptor. This activates the IKK complex, which in turn phosphorylates Iκβ and causes it to degrade (15). Activation of NF-κβ via the canonical pathway triggers production of cytokines in IBD, such as TNF-α and IL-6 (16).

The non-canonical pathway depends upon activation of NF-κβ inducing kinase (NIK); this involves phosphorylation and subsequent activation of the IKKα complex by NIK. Activation leads to phosphorylation of p100 by IKKα, which in turn results in the formation of the p52/RelB active heterodimer. The p52/RelB active heterodimer is then translocated to the nucleus. Induction of the non-canonical pathway occurs through multiple factors, such as, IL-1β lymphotoxin-α, and BAFF. IκBα mediates the turn-off inhibitory signal for NF-κβ by binding with nuclear NF-κβ complexes and transferring them back to the cytoplasm (6). Several genes involved in the non-canonical pathway were significantly higher in diseased tissue of IBD patients vs. adjacent healthy areas and healthy controls (17).

Microbe-associated molecular patterns (MAMPs), damage-associated molecular pattern molecules (DAMPs), cytokines, oxidative stress, bacteria, viruses, and ischemia stimulate and activate the NF-κβ pathway (6).

## NF-κβ in IBD

There is a pathological shift in gut homeostasis in IBD that activates NF-κβ, which in turn further propagates inflammation (8). IBD patients had high levels of NF-κβ, and biopsies of inflamed regions showed a significantly higher number of NF-κβ positive cells compared to normal regions (18). NF-κβ activation has been linked to disease phenotype in CD patients, with high NF-κβ levels correlating with increased ileocolonic and less perianal involvement (19). While NF-κβ is closely linked to IBD, much remains unknown about the specific mechanisms of involvement in disease pathogenesis. Below are a few examples of what is known and how this could impact IBD.

### Cell-specific factors affecting NF-κβ in IBD

Expression and activation of NF-κβ is greatly increased in the gut of IBD patients and is largely cell-specific. NF-κβ subunit p65 levels were found to be higher in the lamina propria of biopsy specimens from CD patients as compared to UC patients and controls (20). NF-κβ is involved in the induction and regulation of many cytokines, including IL-6, TNF-α, IL-1β (21), and IL-12 (9). IL-12 plays an important role in augmenting the differentiation of Th-1 helper cells, and other cytokines, such as TNF-α and IL-23 are also involved in this phenomenon that is critical for inflammation propagation (22, 23). Thus, the effect of NF-κβ on IBD is paramount, as TNF-α is involved in exerting extensive damage to the mucosa and extracellular matrix by being involved in the regulation of, and increasing levels of matrix metalloproteinase (24). In addition, it was found that NF-κβ was induced by IL-6 in colonic epithelial cells and caused an increase in the expression of intercellular adhesion molecule-1 in epithelial cells, which is essential for recruiting neutrophil granulocytes to places of inflammation (25). CD40L induced NF-κβ activation in fibroblasts of colonic epithelial cells, which in turn augmented the expression of IL-6 and IL-8 (26). The IL-6-STAT3 pathway is activated in pediatric IBD (27).

Several cytokines that are increased in IBD and contribute toward inflammation are especially relevant to children. For example, IL-6 is increased in intestinal lamina propria biopsies and serum of pediatric IBD patients (28). In children with IBD, low bone mineral density is attributed to high levels of IL-6 (29). Similar to adults there is an increase in TNF-α levels in the terminal ileum of pediatric CD patients (12). IL-7, IL-1β, IL-5, IL-16, interferon (IFN)-γ-inducible protein-10, leukemia inhibitory factor, monokine induced by IFN-γ, IFN-α2, and IFN-γ were also found to be increased in serum of pediatric IBD patients as compared to healthy control patients, whereas, IL-17, macrophage inhibitory protein-1β, and IL-2 were decreased; many of these cytokines are regulated by NF-κβ-related pathways (30). This imbalance in cytokine regulation indicates the need for further exploration of NF-κβ-related inflammatory pathway in pediatric IBD, as their role in propagating inflammation remains unclear.

Along with its association with inflammation, evidence suggests that NF-κβ has an anti-inflammatory role as well, as seen by increased intestinal inflammation, apoptosis, and reduced antimicrobial peptides in a NEMO-deficient epithelial cell mouse model (31). Similarly, NF-κβ RelA intestinal cell conditional knock-out mice were susceptible to develop DSS-induced colitis (32). These examples highlight the multifaceted, complex nature of innate immune control in the gut.

### Regulation of NF-κβ through A20

A20 is a cytoplasmic protein that acts as a significant inhibitor/regulator of NF-κβ-induced inflammation (33). A20 plays an important role in counter-regulating inflammation in the gut, as shown by the presence of damaged intestinal epithelium and increased apoptosis after intestinal epithelial cells-specific A20 knock-out mice were treated with TNF-α (34). A20 is an important inhibitor of TNF-α-induced NF-κβ inflammation (35). A20 also suppresses CD40 and IL-1, and pattern recognition receptors induced NF-κβ-mediated inflammation (36). A20 expression is increased in pediatric IBD patients with a simultaneous reduction in A20 protein levels, possibly due to destabilization of A20-chaperone factors in IBD (12). Genome-wide association studies (GWAS) have shown linkage between A20 and IBD (11). In adult IBD patients, A20 profiling has shown varying correlations with disease phenotype and severity. A20 expression was low in the colonic and terminal ileum (TI) mucosa (13) and was found to be high in colonic biopsies of adult UC, but not CD patients (37).

### Microbial regulation of NF-κβ

Gut commensals are integral to homeostasis and many regulatory functions, interacting with the mucosal immune system. As such, commensals are heavily involved in anti-inflammatory activities targeted toward NF-κβ in the gut, such as inhibition of NF-κβ activity through peroxisome proliferator activated receptor-γ (PPAR-γ) by *Bacteroides thetaiotaomicron*, which in turn suppresses transportation of the NF-κβ subunit RelA into the nucleus (38). *Bifidobacterium infantis* downregulates NF-κβ activity induced by LPS (39). *Lactobacillus Casei* counteracts inflammation induced by *Shigella flexneri* infection that causes increased transcription of inflammatory cytokines by acting on pathways that stabilize Iκβ and hence prevents translocation of NF-κβ to the nucleus (40). Microbial dysbiosis is a prominent factor involved in IBD and contributes toward inflammation.

### Specific relevance of NF-κβ to pediatric IBD

IBD in children has been subclassified into different categories by age; above or below 10 years, very early onset IBD (VEOIBD) in children < 6 years, and infantile IBD in children < 2 years (41). Pediatric IBD differs from adult IBD in many aspects. Positive family history of IBD is more often the case in pediatric cases vs. in adults. Most often, at the initial stages, IBD occurs in the colon in young children, whereas in adults, small bowel is usually involved. Young children with Crohn disease have more colonic involvement than adults do. Pediatric IBD is more often refractory to medical and surgical treatments commonly used for management of IBD in older patients (42). The proportion of monogenetic causes of IBD-like presentation is highest in the VEOIBD and infantile groups and genetic defects that control NF-κβ, such as variations in TRIMM22, appear to be especially relevant in children (5). Defects and variations in IL-10 and IL-10 receptor are also significant in children with VEOIBD (43, 44). Alterations in other genes, such as, *LRBA* (45), *XIAP* (46), *and TTC7A* (47) is associated with a high risk of developing IBD mostly in childhood (48), but also in adults.

## Involvement of current IBD treatments in NF-κβ pathway

Changes in expression of NF-κβ and associated factors have been observed with several treatments for IBD. In IBD patients treated with corticosteroids, colonic epithelial, mononuclear, and endothelial cells had significantly less nuclear NF-κβ-p65 levels than cells from untreated patients. Corticosteroids increase the expression of IκBα, which retains NF-κβ in the cytoplasm and interacts physically with p65, thus preventing the activation of NF-κβ (49). However, prolonged use of corticosteroids affects linear growth and has the potential to cause hypertension, osteopenia, and increased susceptibility to infection (50).

Sulfasalazine was found to suppress IKKα and IKKβ, which in turn inhibit NF-κβ (51)

*In vitro* experiments revealed that when NF-κβ activation induced by TNF was suppressed by methotrexate, it appeared to be through prevention of phosphorylation and degradation of IκBα, which retains NF-κβ in the cytoplasm and interact physically with p65, thus preventing the activation of NF-κβ (52).

Infliximab treatment caused an increase in IκBα and IκBγ in colonic biopsies; this then inhibits NF-κβ activation and helps in maintaining remission in pediatric patients (53).

Exclusive enteral nutrition (EEN) has been proven to induce clinical remission and lead to mucosal healing in pediatric CD with matching or even superior efficacy to that of corticosteroids (54, 55). Although the use of EEN has been adopted across the globe (56), the mechanism of action remains unclear (54). Increased attenuation of inflammation was also observed in murine models of DSS-colitis along with suppression of TNF-α, IL-6, and IL-8 in colonic biopsies with administration of a novel nutritional polymeric formula. *In vitro* experiments also showed suppression of genes associated with the NF-κβ pathway including, TNF, TNFSF10, NF-κβ1, and RELB with polymeric formula (57). Arginine and glutamine present in polymeric formula suppress phosphorylation involved in the NF-κβ and P38 pathways preventing NF-κβ activation (58). Curcumin, glutamine, and arginine together suppressed IL-8, raising the option of addition of curcumin to polymeric formula to suppress inflammation in IBD (55).

Thus, although current standard therapies for IBD do exert an effect on NF-κβ to some extent, through its associated factors, additional therapies are required for better control of NF-κβ-associated inflammation, Furthermore, much of the published work was done in *in vitro* settings, which is a controlled environment and is starkly different than the actual gut microenvironment where multiple factors are simultaneously at play. Therefore, it is important to focus on the development of a translational approach to develop additional therapies that impact NF-κβ, as described in the following section.

## Potential treatments targeted to modify NF-κβ regulatory and associated factors

Given the pathogenic role of NF-κβ in IBD pathogenesis, it would be attractive to reduce NF-κβ activity by manipulating its regulation. This could be achieved by either directly suppressing NF-κβ or indirectly, by enhancing factors that regulate it. The function of proteasomes differs amongst CD patients and healthy individuals as evident by an increased conversion of the precursor p105 toward its active form, p50 in CD patients (59). Proteasome inhibitors designed for proteasomes that convert the p105 precursor toward active p50 and enhance NF-κβ activation can be beneficial in breaking this pathway. Animal studies have reported successful treatment of TNBS-induced colitis with p65 antisense oligonucleotides that directly target NF-κβ proteins and block them, thus inhibiting the activation of NF-κβ (60). Similar studies have not yet been done in humans, highlighting the importance of early stages of developing drugs focused on enhancing immune regulation, in contrast to current mostly immunosuppressive approaches.

Alterations in gut microbial composition in IBD has been reported by many studies (61) and focus on future targeted microbe-altering therapies is under consideration (62). Fecal microbial transplant (FMT) has been used as a treatment for IBD, but results reported by different studies are variable (63, 64). While studies have shown that gut microbes do exert an inhibitory action on NF-κβ, experimental approaches for studying the mechanism of action of microbes toward NF-κβ in models of IBD and gut environment, such as, organoids derived from IBD patients, need to be developed. As “designer microbes” have been developed to induce immune regulation (through secretion of IL-10, for example) (65), it would be attractive to use microbes to directly regulate the NF-κβ pathway.

Treatments to prevent A20 degradation and administration of factors that stabilize it might be beneficial. A striking correlation was found between A20 and anti-TNF therapy within a Danish cohort of IBD patients, where functional polymorphisms in A20 were predictive of response to anti-TNF therapy (66). Probing another cohort revealed a correlation between *A20* SNPs and response to anti-TNF therapy (67). These findings suggest the possibility of using polymorphisms in *A20* as a genetic biomarker, a venue that should be explored further for practical application. Post-translation modification highly impacts the stability of A20. IKKβ, a kinase required to activate the NF-κβ pathway is also involved in the phosphorylation of A20 at the serine 381 (S381) site, and thus helps A20 to stabilize and stop NF-κβ signaling. As phosphorylation of A20 by IKKβ occurs in response to LPS and TNF (68), presence of IKKβ in inflammation could be a counteractive action to combat NF-κβ induced inflammation too, in addition to stimulating it. Perhaps the key is in studying the conditions that help IKKβ stimulate NF-κβ and altering them.

Paracaspase MALT-1 regulates T cell receptor signaling to NF-κβ and is essential for T cell activation. A20 is directed to a complex of MALT-1 and Bcl-10 upon T-cell receptor stimulation, resulting in its cleavage and rendering it unable to stop the NF-κβ signal (69). Thus, drugs that target MALT-1 can also have an indirect affect on A20, by removing MALT-1 from the cellular environment.

Interaction of A20 with other proteins, such as Tax1 binding protein 1 (TAX1BP-1) and A20-binding inhibitor of NF-κB activation 1 (ABIN-1) helps in attenuating inflammation. The ABIN family of proteins negatively regulate NF-κβ; they are ubiquitin binders and attach to NEMO (NF-κβ essential modulator complex; the IKK complex) (70). ABIN-1 aids A20 to attach to the IKK/NEMO complex and exert its deubiquitinating process (71). The expression of ABIN-1 is dependent upon NF-κβ (72). TAX1BP-1 is also involved in inhibition of NF-κβ-induced inflammation (73) and recruits A20 to the polyubiquitin chains, where A20 breaks and interrupts the IKK complex (74). Ensuring constant presence and stability of TAX1BP-1 and ABIN-1 therefore can play a very important role in suppressing NF-κβ induced inflammation.

As it was reported that CD patients with high NF-κβ levels had more ileocolonic disease and less perianal involvement than those with normal NF-κβ activity (19), it is important to confirm in larger cohorts whether NF-κβ activity is indeed site-specific and correlates with disease status. This could especially be important for pediatric patients, as IBD can possibly be better controlled in early stages of diagnosis and at an earlier age, without the presence of other co-morbidities.

**Figure 2:**
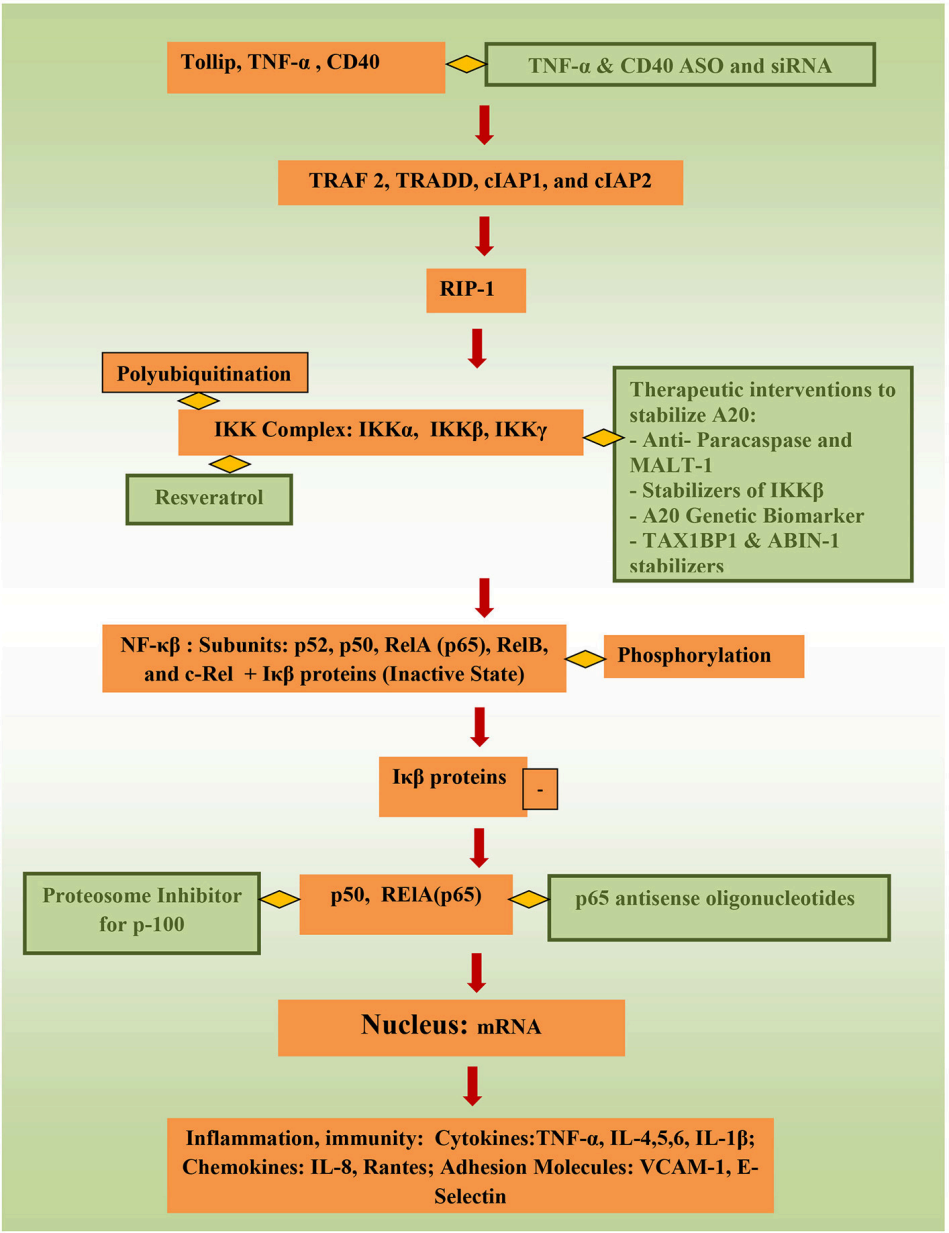
Potential therapeutic pathways for NF-κβ attentation (therapeutic interventions are shown in green).

Targeting controllers of NF-κβ to attenuate its activity directly has tremendous potential to suppress inflammation. Stability of tollip, protein that inhibit inflammation by preventing IL-1 interaction with IL-1Rs, inhibit IRAK phosphorylation and inhibit TLR-2 and TLR-4 mediated inflammation could be of benefit as well (75). In animal models, TNBS-induced colitis and DSS-induced colitis were attenuated through targeting NF-κβ 65 (60, 76), and NF-κβ 65 antisense oligonuceotides (ASO) are undergoing clinical trials currently. Better control of TNF-α (6), which activates the canonical pathway through Toll-like receptors (TLRs), through ASOs and siRNA (small interfering RNA) can be of significance important in precision therapy. Using phosphorothioate ASOs for CD40 also has anti-NF-κβ potential (77, 78). Resrevatrol, an immunomodulator and anti-cancer agent has been found to suppress p65 and IKKβ (79). Figure 2 illustrates the connection between factors affecting NF-κβ and potential therapeutic models that could be relevant to pediatric IBD.

Current IBD therapy is mostly aimed at sustaining immunosuppression. A definite cure is yet to be found. The aim of IBD treatment is induction and maintenance of remission, and prevention of flares and complications. Balance between drug safety and efficacy is a therapeutic challenge, as current medications have serious side effects, emphasizing the need of development of precision therapy for IBD patients. Current IBD treatments affecting NF-κβ are described in Table 1. Potential future treatments for IBD related to NF-κβ are described in Table 2.

**Table 1:** Current IBD Treatments Affecting NF-κβ.

**Treatment**	**Mechanisms of Action**	**References**
Corticosteroids	Increase the expression of IκBα and prevent the activation of NF-κβ	(49)
Sulfasalazine	Suppresses IKKα and IKKβ and inhibits NF-κβ	(51)
Methotrexate	Prevents phosphorylation and degradation of IκBα, retaining NF-κβ in the cytoplasm and preventing its activation	(52)
Infliximab	Increases production of IκBα and IκBγ, which inhibit NF-κβ activation	(53)
Exclusive enteral nutrition (EEN)	Suppression of cytokines TNF-α, IL-6, and IL-8; suppression of related genes: TNFSF10, NF-κβ1, and RELB; prevention of phosphorylation of NF-κβ and p38 pathways	(55, 57, 58)

**Table 2:** Potential future treatments for IBD, related to NF-κβ.

**Treatments**	**Mechanism of Action**	**References**
Proteasome inhibitors	Targeting proteasomes that convert the p105 precursor to active p50 and enhance NF-κβ activation	(59)
p65 antisense oligonucleotides	Directly target NF-κβ proteins and block their action	(60)
Microbial therapy	Use microbes to directly regulate the NF-κβ pathway	(62–65)
A20 stabilizers	Targeting MALT-1; stabilizing IKKβ (phosphorylates A20) and A20 chaperone proteins (ABIN-1, TAX1BP1)	(68–71, 73)

## Future of anti- NF-κβ directed interventions

Treatments directed toward suppressing NF-κβ have a huge therapeutic potential against diseases such as IBD, cancer, and other inflammatory conditions. This is especially relevant in children as controlling the inflammatory cascade at early stages can prevent the devastating long term effects of uncontrolled inflammation as seen in IBD. Systematic *in vitro* and *in vivo* studies in animal models need to be conducted to analyze factors that antagonize/attenuate NF-κβ, and results need to be interpreted with caution; however, in contrast to almost all IBD treatments used today, which suppress the immune response, modulating pathways, for example by enhancing NF-κβ regulating molecules, such as A20, could allow the gut environment to return to homeostasis, possibly without an increase in infection risk. Obviously, before translating potential treatments to treat humans, safety and efficacy of potential therapeutic regimens needs to be established. Development of medications specifically targeting NF-κβ to control its inflammatory activity can be of tremendous benefit to children with IBD, such as early control of symptoms and low risk of immunosuppression that is often associated with current IBD medications.

## Author contributions

All authors listed have made a substantial, direct and intellectual contribution to the work, and approved it for publication.

### Conflict of interest statement

The authors declare that the research was conducted in the absence of any commercial or financial relationships that could be construed as a potential conflict of interest.
